# Supplementary material for: Cytogenetic events in the endosperm of amphiploid Avena magna × A. longiglumis
Source: J Plant Res. 2021 May 31;134(5):1047–60. doi: 10.1007/s10265-021-01314-3 (PMC8364899; doi:10.1007/s10265-021-01314-3)
Supplement: Supplementary file 1 — Supplementary file1 (PDF 389 KB) [file 10265_2021_1314_MOESM1_ESM.pdf]

Supplementary file

Cytogenetic events in the endosperm of amphiploid *Avena magna* × *A. longiglumis*

Journal of Plant Research

Paulina Tomaszewska[1] · Romuald Kosina[2] ·

[1] Institute of Experimental Biology, and [2] Institute of Environmental Biology, University of Wrocław, Wrocław, Poland

Correspondence: R. Kosina, Institute of Environmental Biology, University of Wroclaw, Przybyszewskiego 63, 51-148 Wroclaw, Poland; e-mail: romuald.kosina@uwr.edu.pl

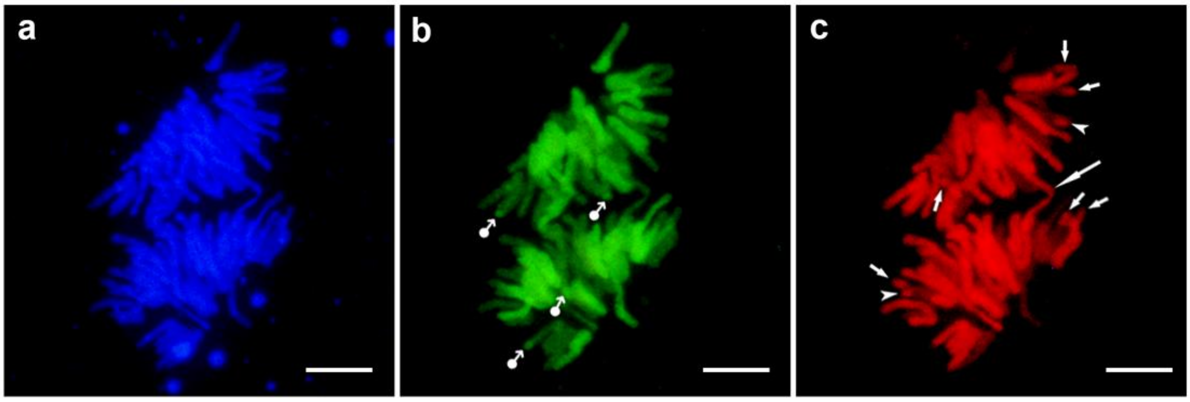

Fig. S1 Chromosome rearrangements in *Avena magna* detected by GISH. Pictures are related to Fig. 2c in the article, showing studied anaphase in three different filters. **a** – DAPI-stained chromosomes; **b** – FITC-detected *Avena nuda* probe (As genome); **c** – TRITC-detected *Avena eriantha* probe (Cp genome). Translocations are marked by dot-arrows for terminal D/C, arrows for terminal C/D, and arrowheads for subterminal C/D. The large arrow indicates the bridge. Scale bars 10 µm
